# PROTAC Bromodomain Inhibitor ARV-825 Displays Anti-Tumor Activity in Neuroblastoma by Repressing Expression of *MYCN* or *c-Myc*

**DOI:** 10.3389/fonc.2020.574525

**Published:** 2020-11-26

**Authors:** Zhiheng Li, Su Lin Lim, Yanfang Tao, Xiaolu Li, Yi Xie, Chun Yang, Zimu Zhang, You Jiang, Xianbing Zhang, Xu Cao, Hairong Wang, Guanghui Qian, Yi Wu, Mei Li, Fang Fang, Ying Liu, Mingcui Fu, Xin Ding, Zhenghong Zhu, Haitao Lv, Jun Lu, Sheng Xiao, Shaoyan Hu, Jian Pan

**Affiliations:** ^1^Institute of Pediatric Research, Children’s Hospital of Soochow University, Suzhou, China; ^2^Department of Pathology, Brigham and Women's Hospital, Harvard Medical School, Boston, MA, United States; ^3^Department of Internal Medicine, Saint Michael’s Medical Center, Newark, NJ, United States; ^4^Department of Hematology, Children’s Hospital of Soochow University, Suzhou, China; ^5^Department of Pediatric Surgery, The First People’s Hospital of Kunshan, Suzhou, China; ^6^Department of Pediatric Surgery, Children’s Hospital of Soochow University, Suzhou, China; ^7^Department of Pathology, Children’s Hospital of Soochow University, Suzhou, China; ^8^Department of Neonatology, Children’s Hospital of Soochow University, Suzhou, China; ^9^Department of Burn and Plastic Surgery, Children’s Hospital of Soochow University, Suzhou, China; ^10^Department of Cardiology, Children’s Hospital of Soochow University, Suzhou, China

**Keywords:** neuroblastoma, BRD4, *MYCN*, *c-Myc*, ARV-825

## Abstract

Neuroblastoma (NB) is one of the most common solid tumors in childhood. To date, targeting *MYCN*, a well-established driver gene in high-risk neuroblastoma, is still challenging. In recent years, inhibition of bromodomain and extra terminal (BET) proteins shows great potential in multiple of *Myc*-driven tumors. ARV-825 is a novel BET inhibitor using proteolysis-targeting chimera (PROTAC) technology which degrades target proteins by the proteasome. In this study, we investigated the effect of ARV-825 in neuroblastoma *in vitro* and *in vivo*. Our results showed that ARV-825 treatment robustly induced proliferative suppression, cell cycle arrest, and apoptosis in NB cells. Moreover, ARV-825 efficiently depleted BET protein expression, subsequently repressing the expression of *MYCN* or *c-Myc*. In the NB xenograft model, ARV-825 profoundly reduced tumor growth and led to the downregulation of BRD4 and *MYCN* expression in mice. Taken together, these findings provide evidence that PROTAC BET inhibitor is an efficient way to achieve *MYCN*/*c-Myc* manipulation, and ARV-825 can be used as a potential therapeutic strategy for the treatment of neuroblastoma.

## Introduction

Neuroblastoma (NB) is a common pediatric malignancy originating from the embryonic sympathetic nervous system, of which 90% of cases are diagnosed under age 5 (1, 2). Although the low and intermediate-risk patients generally exhibit favorable outcomes, the five-year event-free survival rate for the high-risk group is less than 50% (2). *MYCN* is regarded as one of the most commonly validated genes implicated in NB tumorigenesis, which is amplified in about 50% of high-risk cases (3). *MYCN* amplification strongly correlates to an undifferentiated, aggressive phenotype and indicates an adverse prognosis (4). However, targeting the *Myc* family protein, including N-Myc, is still challenging due to the lack of pockets that could be targeted directly with small molecules (5). For this reason, indirect targeting strategies are currently being explored to achieve Myc inhibition, which has become a promising therapeutic approach for these *Myc*-driven cancers.

Manipulation of epigenetic modifiers, such as inhibiting the bromodomain and extra terminal (BET) proteins that link chromatin markers to activate *Myc* transcription, has been proven to be an effective way to block *Myc* expression indirectly (2). The BET family, which is composed of BRD2, BRD3, BRD4, and BRDT, can recognize and bind acetylated lysine modifications of histones. They play a fundamental role in transcription activation. Among them, BRD4 is the most characterized member that enriches at the super enhancer region at *Myc* locus, resulting in genome-wide regulation of *Myc*-dependent target genes (6, 7). In addition, targeting BRD4 by BET inhibitor displaces BRD4 from the *MYCN* promoter region and downregulates *MYCN* expression in neuroblastoma cells, establishing BRD4 as a transcriptional regulator of *MYCN* (8).

Over the last decade, much effort has been made on the development of small molecular inhibitors targeting the BET family. The anti-tumor activity of the first-generation BET inhibitor JQ1 was first demonstrated in NUT midline carcinoma harboring a *BRD4-NUT* fusion gene (9). Thereafter, the efficacy of JQ1 was also evaluated in a broad range of tumors, including hematological malignancy (6, 10) and other solid tumors (11–13), showing anti-proliferative and pro-apoptotic activity. Many other BET inhibitors (BETi) were developed and demonstrated a promising anti-tumor effect. In the recent years, several BETi have been introduced into clinical trials to determine their effectiveness for human cancer treatment (14). OTX015, a JQ1 analog compound under clinical phase I trials for patients with solid tumors and hematologic malignancies, exhibits great efficacy in a broad range of tumors (15–18).

In neuroblastoma, Alexandre Puissant et al. reported that *MYCN*-amplified neuroblastoma show sensitivity to BET inhibitor JQ1. JQ1 treatment leads to cell cycle arrest and promotes apoptosis in *MYCN*-amplified NB cells (8). In addition, JQ1 was shown to promote neural differentiation *in vitro* and *in vivo* (19). In another preclinical model, OTX015 was shown to be effective against both *in vitro* and *in vivo MYCN*-driven neuroblastoma model (20). The BET inhibitor BMS-986158 is currently under clinical trial in pediatric cancer, including neuroblastoma (NCT03936465). Furthermore, some studies described a combination therapy of BETi with other drugs has a synergistic effect against NB tumor progression (21, 22).

Although previous observations have shown promising results of BETi in interfering with BRD4 function, the effect of BETi such as JQ1 and OTX015 are reversible which causes the re-accumulation of BRD4 protein and incomplete suppression of *MYC* (23). This has inspired the generation of novel BRD4 targeting molecules using PROTAC technology (24). Proteolysis-targeting chimeras (PROTACs) are hetero-bifunctional small molecules employing E3 ligase ligands, fused via a ﬂexible chemical linker to a ligand that recognizes the target protein. Such molecules can recruit the target protein to the E3 ligase, elicit ubiquitination of the target protein which leads to its degradation through the ubiquitin-proteasome system (UPS) (25, 26). Compound inducing degradation of BET proteins has shown superior antineoplastic effects over BETi, suggesting a better way to target BET members (27).

ARV-825 is a newly developed inhibitor using PROTAC technology, which conjugating OTX015 with an E3 ligase cereblon (CRBN). Administration of ARV-825 renders recruitment of BRD4 to cereblon and result in a rapid, efficient, and prolonged BRD4 degradation (24). Sujan Piya et al. showed that BRD4 degradation by ARV-825 leads to increased ROS generation, thus elevating the oxidative stress in AML cells. More importantly, ARV-825 treatment decreases the stem cell population and prolonged survival in the AML-PDX model (28). Our previous study and Zhang et al. have both demonstrated that ARV-825 has promising activity against pre-clinical models of multiple myeloma by degrading BRD4 protein and subsequently leads to downregulation of BRD4 target genes, including *MYC* (29, 30). Yet, the antitumor potency of ARV-825 has not been elucidated in neuroblastoma.

In this study, we examined the effect of PROTAC BET inhibitor ARV-825 on neuroblastoma cell lines and xenograft mice model. Our results showed that ARV-825 treatment significantly inhibited cell growth, cell cycle progression, and induced apoptosis in NB cells. Furthermore, ARV-825 reduced tumor growth in xenograft mice model. ARV-825 exerted its effect by degrading BET proteins and subsequently suppressing the *MYCN* or *c-Myc* expression in NB cells. Our studies demonstrated the preclinical efficacy of ARV-825 as a novel therapeutic strategy for clinical NB treatment.

## Methods and Materials

### Cell Culture

The neuroblastoma cell lines [SK-N-SH, SH-SY5Y, IMR-32 and SK-N-BE(2)] were purchased from the cell bank of the Chinese Academy of Science within 5 years. All cell lines were verified by short tandem repeat analysis in the year of 2018. Cells were maintained in DMEM or MEM medium (Thermo Fisher Scientific) containing 10% FBS (Biological Industries, CT, USA) and 1% penicillin-streptomycin (MilliporeSigma, MA, USA) at 37°C with 5% CO_2_ and tested free of *Mycoplasma* routinely.

### Plasmids and Reagents

The short hairpin RNA (shRNA) targeting CRBN (Sequence is available in **Supplement Material 1**) in pLKO.1 lentiviral vector and pLX304-CRBN-V5 vector (PMID: 29764999) were a kind gift from Dr. X. Liang (Cancer Science Institute, Singapore). For lentivirus preparation, the envelop plasmid and packaging plasmid was purchased from Addgene (pMD2.G: #12259; psPAX2: #12260). ARV-825 was purchased from MedChemExpress (NJ, USA).

### Tissue Microarray

The tissue microarray containing 27 NB patients’ samples and 5 peripheral nerve tissues was purchased from Biomax, lnc. (Derwood, MD, USA; Cat: MC642). The immunohistochemistry staining was performed as previously described (31). The primary antibody against BRD4 (Cat: ab128874, Abcam) was used with corresponding concentration (1:200) according to the manufacturer's recommendations. Rabbit specific HRP/DAB detection kit (Cat: ab64261, Abcam) was used following standard protocol. The staining results of each tissue section were observed under the Olympus BX41 imaging system and assessed by 2 pathologists separately. The total scoring (TS) results were scored by multiplying the percentage of positive cells (P) by the intensity (I). Formula: TS = P x I.

### Lentivirus Preparation and Infection

pMD2.G, psPAX2, and the transfer plasmid were co-transfected into 293FT cells with PEI (linear MW 25,000 Da, 5 mg/ml, pH = 7.0) (Cat: 23966-1, Polysciences, Warrington, PA, USA). Complete culture medium change was performed 6–8 h post-transfection. The viral supernatant was harvested at 48 h post-transfection and filtered through a 0.45 μm filter. Then, prepared lentivirus was aliquoted immediately and stored at -80°C. NB cells were infected with lentivirus in the presence of 10 μg/ml polybrene (Sigma-Aldrich) for 24 h. Stable cell lines were generated by puromycin or blasticidin (Sigma-Aldrich) selection.

### Cell Viability Assay

NB cells were seeded in 96-well plates at a density of 2x10^4^ cells per well. Allowing to attach overnight, cells were treated with different concentrations of ARV-825. After 72 h drug treatments, cell viability was determined by cell counting kit-8 (CCK8) assay (Dojindo Molecular Technologies, Tokyo, Japan) as described before (32). The absorbance at 450 nm was measured using a microplate reader (Thermo Fisher). Each concentration was performed in triplicate and repeated at least in three independent experiments. The IC50 of ARV-825 was calculated by Graph Prism software 8.3.0 (GraphPad-Prism Software Inc., San Diego, CA, USA).

### Cell Cycle Analysis

NB cells were trypsinized, washed, and fixed in 70% ethanol at 4°C overnight. Cells were washed with cold phosphate-buffered saline (PBS), incubated with 1.5 µM propidium iodide (PI, cat. P4170; Sigma-Aldrich, St. Louis, MO, USA) solution containing RNase A (25 µg/ml) at room temperature for 1 h. After measurement by flow cytometry on a Beckman Gallios™ Flow Cytometer (Beckman, Krefeld, Germany), cell cycle distribution was analyzed by MultiCycle AV DNA analysis software (Verity Software House, Topsham, ME, USA).

### Cell Apoptosis Assay

Cell apoptosis was determined as previously described (33). Briefly, NB cells were incubated with ARV-825 at indicated concentrations. After 72 h incubation, cells were harvested and washed with cold PBS. Suspended in the 1× binding buffer, cells were stained by FITC-Annexin V antibody and PI solution according to the manual of the FITC-Annexin V apoptosis kit (cat. 556420; BD Biosciences, Franklin Lakes, NJ, USA). Cell apoptosis was analyzed by flow cytometry on a Beckman Gallios™ Flow Cytometer (Beckman, Krefeld, Germany).

### RNA Preparation, Real-Time PCR Expression Analysis

Total RNA was isolated from cells using the RNeasy Mini Kit (cat. 74104; Qiagen, Germany). First-strand cDNA was synthesized from 2 μg of total RNA as template, 500 ng of six random primers (Promega, USA), 200U of M-MLV Reverse transcriptase (Promega, USA), and 20U of RNase inhibitor (Thermo Fisher Scientific, MA, USA) in a total volume of 25 μL. Quantitative real-time polymerase chain reaction analysis was conducted with LightCycler® 480 SYBR Green I Master mix (cat. 04707516001; Roche, Penzberg, Germany) on a Light cycler 480 Real-Time System (Roche, Penzberg, Germany) according to the standard protocol. Quantitative mRNA expression was calculated using the *Ct* method and GAPDH expression as an internal reference. Real-time PCR primers are listed in **Supplement Material 1**.

### Western Blot Analysis

Whole-cell extracts were prepared by incubating cells in RIPA buffer supplemented with protease and phosphatase inhibitor cocktail (Roche, Penzberg, Germany) for 30 min on ice. The supernatant was collected by centrifuge and protein concentration was quantified using the Pierce BCA Kit (Thermo Fisher Scientific). Blotting was conducted as previously described (33). Primary antibodies against the following proteins were used: BRD2 (Cat: 5848s, 1:1,000, Cell Signaling Technology), BRD3 (Cat: 11859-1-AP, 1:1,000, Proteintech), BRD4 (Cat: 13440s, 1:1000, Cell Signaling Technology), CRBN (Cat: HPA045910, Sigma-Aldrich), *c-Myc* (Cat: 9402, 1:1,000, Cell Signaling Technology), *MYCN* (Cat: sc-53993, 1:1,000, Santa Cruz Biotechnology), cleaved-Caspase 3 (Cat: 9664, 1:1,000, Cell Signaling Technology), PARP (Cat: 9542, 1:1,000, Cell Signaling Technology). β-actin (Cat: A5441, 1:5,000, Sigma-Aldrich) or GAPDH (1:2,000; MA3374, Millipore) were used as a reference protein. The horseradish peroxidase-conjugated secondary antibodies Peroxidase AffiniPure Goat Anti-Mouse IgG(H+L) (Cat: 115-035-003) and Goat Anti-Rabbit IgG(H+L) (Cat: 111-035-003) were purchased from Jackson ImmunoResearch Laboratories, INC. The bands were visualized by an ECL detection kit (Pierce, Rockford, IL, USA) using LAS 4010 imaging system (GE Healthcare Life Sciences, Little Chalfont, UK).

### *In Vivo* Xenografts

All animal procedures in this study were approved and licensed by the Animal Care and Use Committee at Children’s hospital of Soochow University. Nude mice were obtained from Lingchang BioTech Co., Ltd. (Shanghai, China). Five-week-old male nude mice (n = 8 per group) were injected subcutaneously in the frontier flank with 1×10^7^ SK-N-BE(2) cells. Subcutaneous tumor size was monitored using calipers every 2–3 days. Tumor volume was calculated according to the formula (width × length × height)/2. When the engrafted tumor reached a size of about 100 mm^3^, either 5 mg/kg of ARV-825 or vehicle alone (5% Kolliphor®HS15) were given intraperitoneally every day. Animals were sacriﬁced when tumor size exceeded 1,000 mm^3^, which was defined as the survival endpoint. The xenografted tumors were embedded in parafﬁn. The primary antibody against Ki-67 (Cat: ab15580, Abcam) was used with corresponding concentration (1:500) according to the manufacturer's recommendations. IHC was performed as described above.

### Statistical Analysis

All experiments were independently performed in triplicate at least 3 times. Statistical analyses were performed using GraphPad Prism version 8.3.0 (GraphPad Software, Inc., San Diego, CA, USA). *p* values less than 0.05 were regarded as statistically significant (*p < 0.05, **p < 0.01, ***p < 0.001). Means±Standard Deviation (SD) are shown.

## Results

### High BRD4 Expression Is Associated With Poor Prognosis in NB Patients

**Figure 1:**
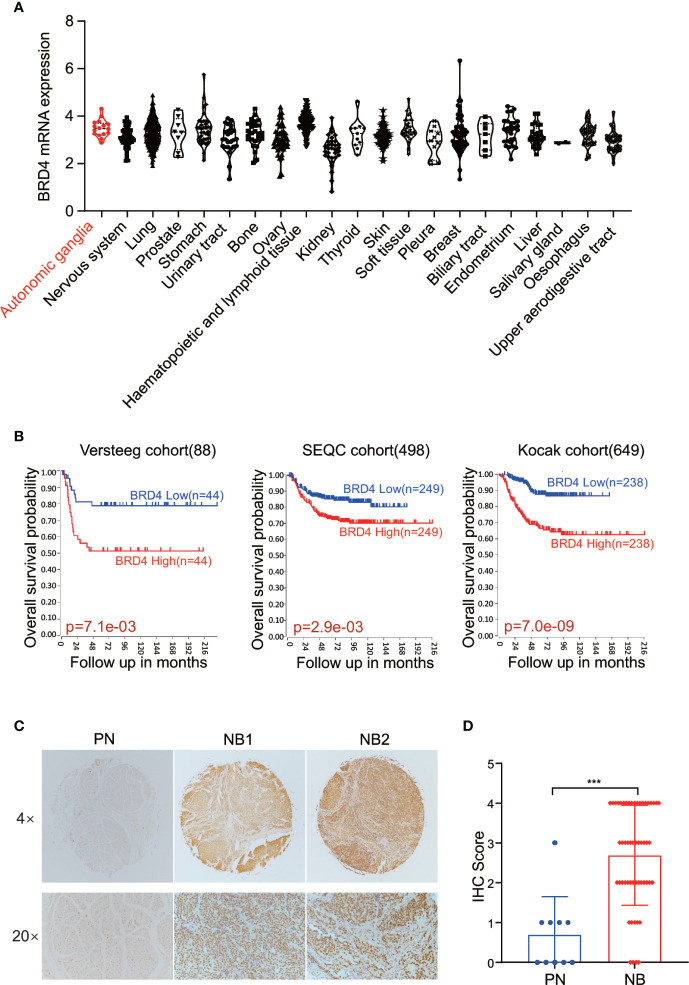
BET proteins are universally expressed in NB cells. **(A)** BRD4 mRNA expression level in a broad range of tumors (generated from Broad Cancer Cell Line Encyclopedia: https://portals.broadinstitute.org/ccle). **(B)** Overall survival curve using public cohorts including 88 NB patients (left), 498 NB patients (middle), and 639 NB patients (right, 173 samples were omitted because of lack of survival data) generated from R2 Genomics Analysis and Visualization Platform (http://r2.amc.nl). Median survival time was used as a cutoff point for categorized as high or low expression. **(C)** Representative images of immunohistochemistry staining showed elevated BRD4 protein expression in NB patients’ samples as compared to peripheral neuron. **(D)** Histologic scores were determined according to the intensity of BRD4 staining. ***p < 0.001. PN, peripheral neuron; NB, neuroblastoma.

First, we sought to analyze the BRD4 expression in different types of tumors. The CCLE (Cancer Cell Line Encyclopedia: https://portals.broadinstitute.org/ccle) displayed the BRD4 mRNA expression profile across different types of cancer cell lines, showing that BRD4 was expressed universally without distinct cancer type specificity (**Figure 1A**). The prognostic significance of BRD4 in NB patients was also evaluated. NB patients from three different cohorts in the R2 platform were used to analyze the association of BRD4 expression level with the overall survival of NB patients. The Kaplan-Meier curves were generated from three public neuroblastoma expression datasets derived from the GEO database, including 88, 498, and 649 NB patients respectively (GEO accession: GSE16476; GSE49710; GSE45547). Median survival time was used as a cutoff point for categorized as high or low expression. As indicated in **Figure 1B**, high BRD4 expression was associated with unfavorable outcome in NB patients.

We further investigated the association of BRD4 expression with NB prognosis in the Kocak cohort containing 649 NB samples by applying the other four cutoff modi provided in the R2 platform. As shown in the **Supplement Figure S1**, no matter which cutoff modus was applied, patients with higher BRD4 expression had worse overall survival rates than those with lower BRD4 expression.

Immunohistochemistry (IHC) was performed to detect BRD4 protein by using tissue microarray which contained 27 NB patients’ samples and 5 peripheral nerve tissues as control. The protein level of BRD4 was compared between neuroblastoma and peripheral neurons. As shown in the **Figures 1C, D**, tissues from NB patients displayed moderate to high nuclear staining of BRD4, while most control neurons were negative. These results indicate that the protein level of BRD4 is significantly elevated in NB samples compared with the control neurons. These results suggest that BRD4 can be used as a potential therapeutic target for neuroblastoma.

### NB Cells Are Sensitive to ARV-825 Treatment

**Figure 2:**
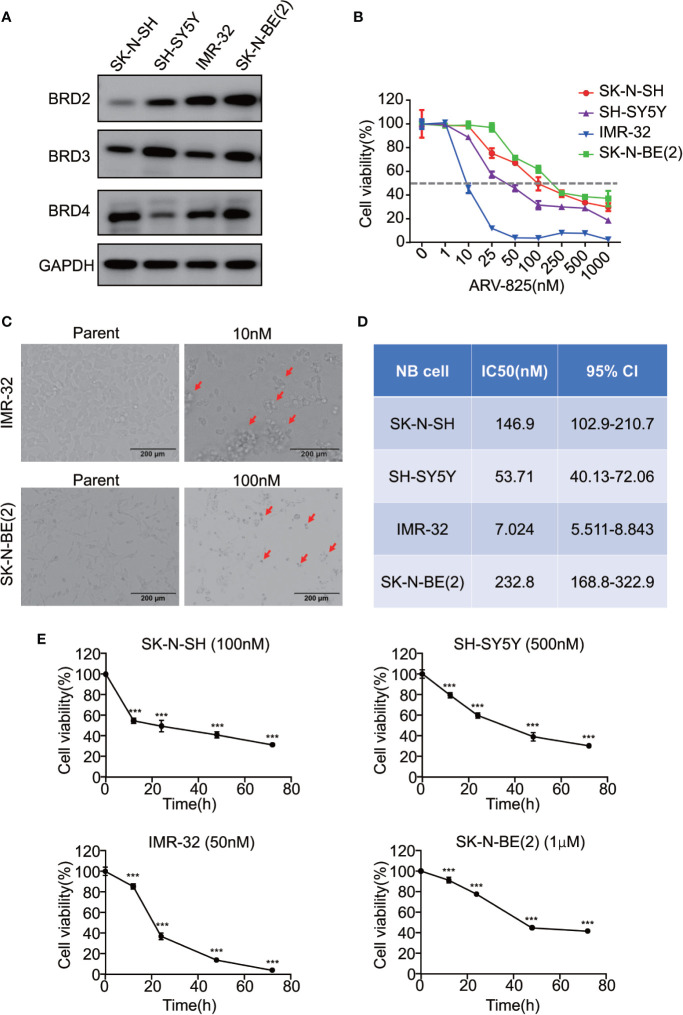
ARV-825 inhibits cell viability in NB cells. **(A)** Western blot analysis showed basal BET protein expression level in NB cells. **(B)** Cell viability of NB cells treated with serial concentrations of ARV-825 for 72 h. The cell viability rate was calculated as a percentage of the DMSO-treated control wells. **(C)** Morphology of IMR-32 (up) and SK-N-BE(2) (down) cells incubated with ARV-825 at indicated concentrations for 72 h (red arrows indicated dead cells after ARV-825 treatment). **(D)** The IC50 value of ARV-825 in different NB cell lines. **(E)** Cell viability of NB cells treated with ARV-825 at various times. (SK-N-SH were treated with 100 nM ARV-825; SH-SY5Y were treated with 500 nM ARV-825; IMR-32 were treated with 50 nM ARV-825; SK-N-BE(2) were treated with 1 μM ARV-825). ***p < 0.001.

Given that the BET family is expressed ubiquitously, except for BRDT, which is only expressed in the testis (14), two *MYCN*- amplified NB cell lines [IMR-32 and SK-N-BE(2)] and two *MYCN* non-amplified NB cell lines (SK-N-SH and SH-SY5Y) were used to study the expression level of BRD2, BRD3, and BRD4. The mRNA expression levels of BRD2, BRD3, and BRD4 were shown in **Supplement Figure S2**. At the protein level, BRD4 protein was abundantly expressed in all four NB cell lines, regardless of the *MYCN* status (**Figure 2A**), indicating that the BET family members are universally expressed in NB cells.

**Figure 3:**
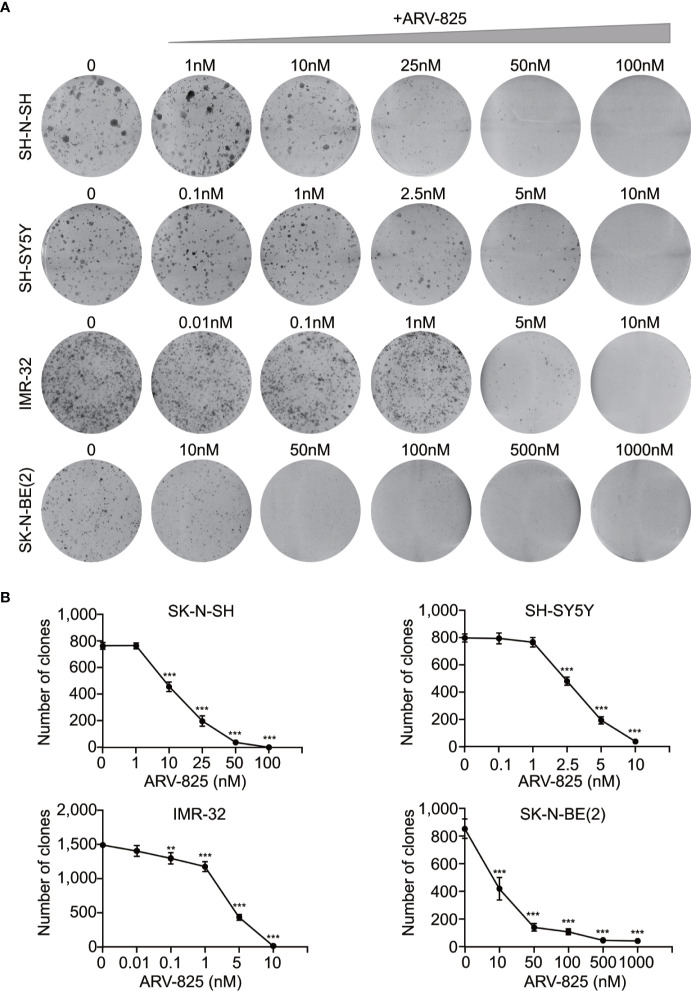
ARV-825 inhibits clonal formation in NB cells. **(A)** Clone formation assay showed increasing doses of ARV-825 inhibited the clonal formation ability in NB cells. **(B)** Clone numbers of NB cells treated with increasing doses of ARV-825. **p < 0.01; ***p < 0.001.

The structure of PROTAC BRD4 inhibitor ARV-825, composed of OTX015 and a CRBN recruiting moiety connected by a “linker”, was shown in **Supplement Figure S3**. In order to evaluate the effect of ARV-825 on NB cell lines, the cells were treated with increasing doses of ARV-825 for 72h. CCK8 assay showed that NB cell viability was reduced in a dose-dependent fashion after ARV-825 treatment (**Figure 2B**). Changes in cell morphology were observed in the ARV-825-treated group, with cells clustered and floating (**Figure 2C**). All of the four NB cell lines were sensitive to ARV-825, with IC50 ranging from 7.024 to 232.8 nM (**Figure 2D**) (SK-N-SH IC50: 146.9 nM; SH-SY5Y IC50: 53.71 nM; IMR-32 IC50: 7.024 nM; SK-N-BE(2) IC50: 232.8 nM). Additionally, ARV-825 treatment also remarkably reduced NB cell growth in a time-dependent manner (**Figure 2E**). The impact of ARV-825 on the long-term proliferation of NB cells was determined by clonal formation assay. As demonstrated in **Figure 3**, ARV-825 effectively suppressed the clonal growth in all four NB cell lines. Collectively, these data suggest ARV-825 exerts a potent anti-proliferative effect in NB cell lines.

### CRBN Expression Is Indispensable to Sensitivity to ARV-825

**Figure 4:**
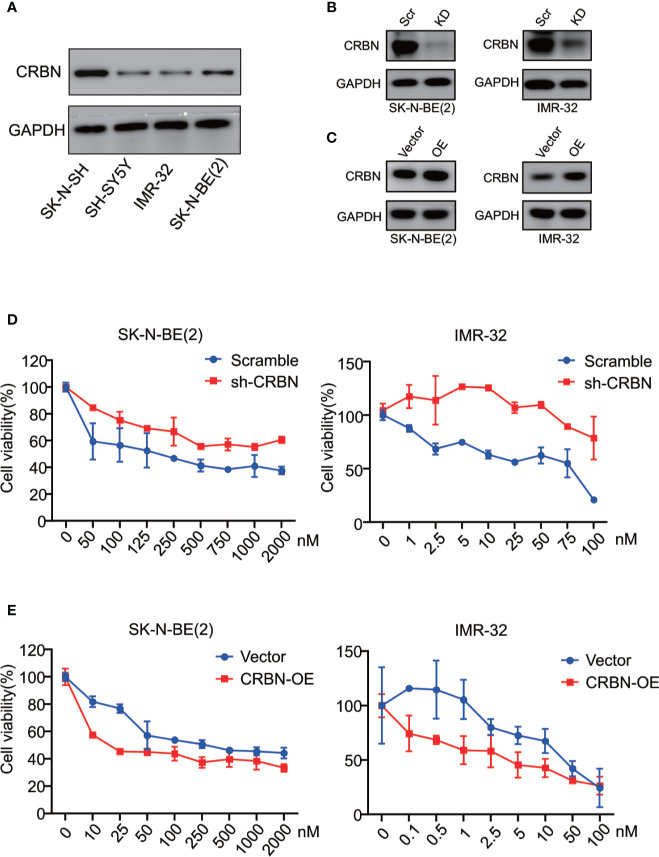
CRBN is indispensable in responsiveness to ARV-825 in NB cells. **(A)** Western blot analysis showed basal CRBN protein level in NB cells. **(B)**Knockdown of CRBN expression by sh-CRBN lentivirus in SK-N-BE(2) (left) and IMR-32 (right) cells. Scr, Scramble; KD, knockdown. **(C)** Overexpressing CRBN in SK-N-BE(2) (left) and IMR-32 (right) cells. OE, overexpression. **(D)** Comparison of sensitivity to ARV-825 of cells transfected by sh-CRBN with cells transfected by sh-scramble in SK-N-BE(2) (left) and IMR-32 (right) cells. **(E)** Comparison of sensitivity to ARV-825 of cells overexpressing CRBN with cells transfected by empty vector alone in SK-N-BE(2) (left) and IMR-32 (right) cells. OE, overexpression.

We previously reported that CRBN mRNA expression levels in different MM cell lines are correlated with their sensitivity to ARV-825 (29). In NB cells, three out of four NB cells have an appreciable expression of CRBN except for IMR-32 cells (**Figure 4A** and **Supplement Figure S4**). Even though IMR-32 cells have a relatively low CRBN expression at both mRNA and protein levels, it is the most sensitive cell line to ARV-825. We further explored whether the efficacy of ARV-825 in NB cells is dependent on CRBN expression. Compared with cells stably transfected with sh-Scramble, knockdown of CRBN expression by using specific shRNA in IMR-32 and SK-N-BE(2) cells partially rescued the anti-proliferative effect of ARV-825 (**Figures 4B, D**). On the contrary, overexpressing CRBN in NB cells significantly increased the sensitivity to ARV-825 in IMR-32 and SK-N-BE(2) cells (**Figures 4C, E**). These observations indicate that CRBN expression is indispensable to the anti-proliferative activity of ARV-825 in NB cells.

### ARV-825 Induces Cell Cycle Arrest and Apoptosis in NB Cells

**Figure 5:**
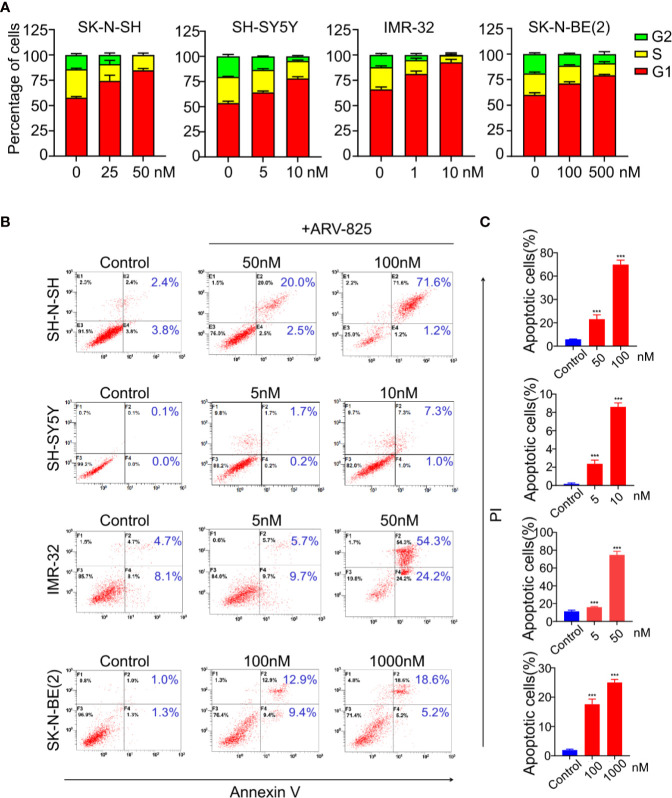
ARV-825 elicits cell cycle arrest and apoptosis in NB cells. **(A)** Cell cycle analysis showed ARV-825 induced an increased proportion of the G1 phase and concurrently decreased S and G2 proportion in NB cells. **(B)** Annexin V/PI staining showed an increased proportion of apoptotic cells in NB cells after treated with ARV-825 at indicated concentrations. **(C)** The proportion of apoptotic cells increased significantly in NB cells treated by ARV-825. ***p < 0.001.

BET family has been well established as cell cycle regulators. Therefore, we examined the effect of ARV-825 on cell cycle in NB cells. Four NB cells were treated with different concentrations of ARV-825 for 24 h. Cell cycle analysis was then performed by PI staining. Exposure to ARV-825 led to an increase in G_1_ phase proportion, accompanied by a decrease in S and G_2_ phase proportion across all the NB cells analyzed (**Figure 5A**). These data reveal that ARV-825 potently triggers G_2_/M cell cycle arrest in NB cells.

**Figure 6:**
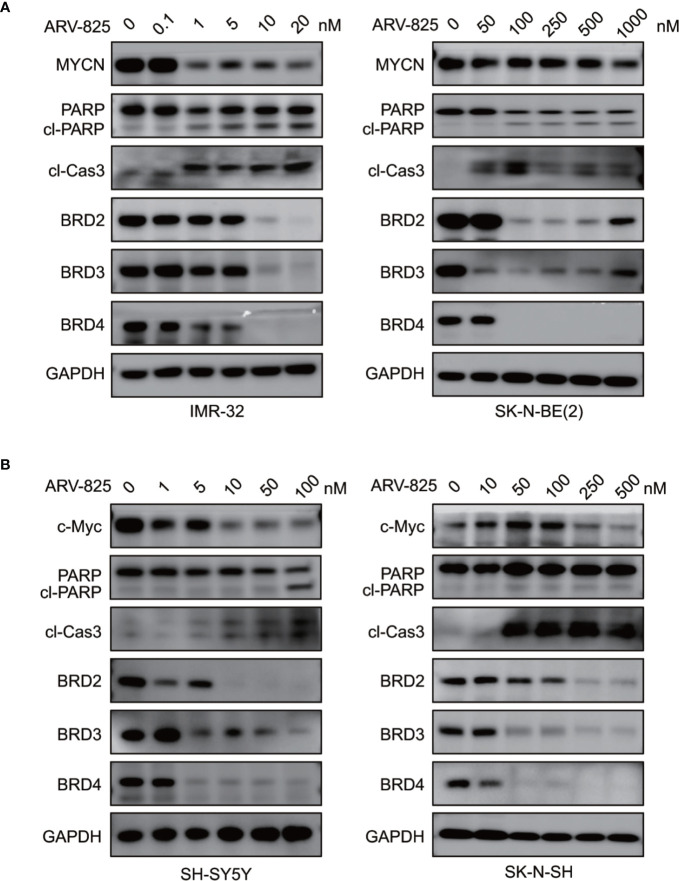
ARV-825 degrades BET proteins and suppresses *MYCN* or *c-Myc* expression. **(A)** Western blot analysis showed that ARV-825 induced BET proteins degradation, PARP and Caspase3 cleavage, and MYCN protein reduction in *MYCN*-amplified NB cells [left: IMR-32 cells; right: SK-N-BE(2) cells]. **(B)** Western blot analysis showed that ARV-825 induced BET proteins degradation, PARP and Caspase3 cleavage, and c-Myc protein reduction in *MYCN* non-amplified NB cells (left: SH-SY5Y cells; right: SK-N-SH cells).

We also examined the impact of ARV-825 on apoptosis in NB cells. The Annexin V/PI staining showed that ARV-825 treatment robustly elicited apoptosis in all cell lines in a dose-dependent manner. The proportion of apoptotic cells increased in the ARV-825-treated group compared with DMSO-treated control cells (**Figures 5B, C**). Western blot analysis confirmed the pro-apoptotic effect of ARV-825 by showing substantial cleavage of PARP and Caspase-3 in all four NB cells in response to ARV-825 treatment (**Figures 6A, B**). These findings indicate that ARV-825 can abrogate cell cycle progression and induce apoptosis in NB cells.

### ARV-825 Degrades BET Protein Expression in NB Cells

As ARV-825 is designed by PROTAC technology which selectively degrades target protein by the ubiquitin-proteasome system, we then further analyzed the BET protein expression following ARV-825 treatment in NB cells. Western blotting was performed and showed that the treatment of four NB cells with serial concentrations of ARV-825 induced sustained degradation of BRD4 protein (**Figures 6A, B**). Other than BRD4, ARV-825 also potently reduced the BRD2 and BRD3 protein expression (**Figures 6A, B**). These data suggest ARV-825 downregulates BET protein expression in NB cells.

### ARV-825 Reduces *MYCN* or *c-Myc* Expression in NB Cells

**Figure 7:**
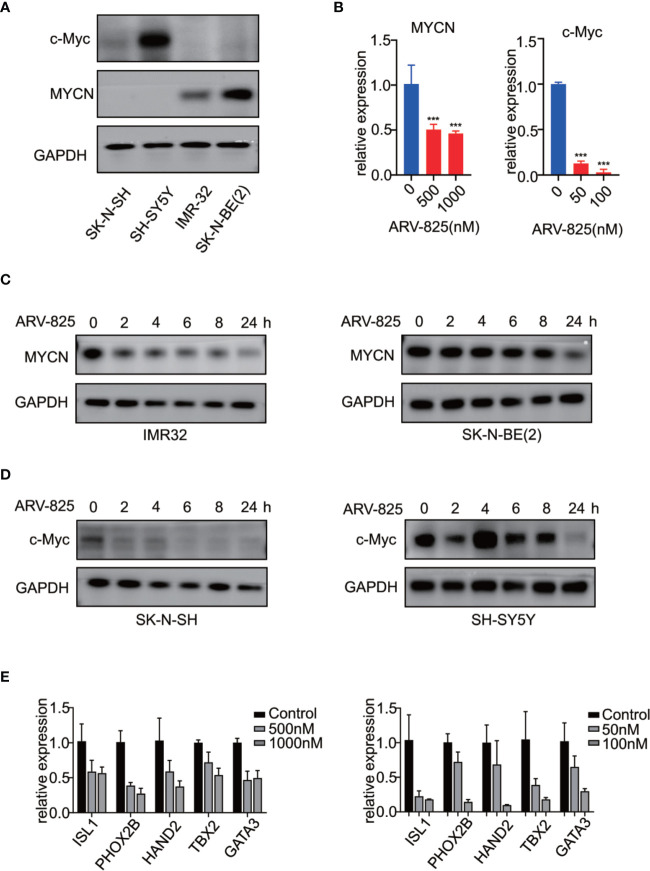
ARV-825 suppresses MYCN/c-Myc expression and *MYCN*-associated super enhancers expression. **(A)** Western blot analysis showed MYCN and c-Myc protein levels in NB cells. **(B)** Real-time PCR analysis showed that ARV-825 induced suppression of *MYCN* and *c-Myc* mRNA relative expression [left: SK-N-BE(2) cells; right: SH-N-SH cells]. **(C)** Western blot analysis showed MYCN protein was downregulated by treatment with ARV-825 at different times in *MYCN*-amplified NB cells [left: IMR-32 cells treated by 10 nM ARV-825; right: SK-N-BE(2) cells treated by 500 nM ARV-825]. **(D)** Western blot analysis showed c-Myc protein was downregulated by treatment with ARV-825 at different times in *MYCN* non-amplified NB cells (left: SK-N-SH cells treated by 50 nM ARV-825; right: SH-SY5Y cells treated by 50 nM ARV-825). **(E)** Transcript level of *MYCN*-associated super enhancers was downregulated following ARV-825 treatment in SK-N-BE(2) cells (left) and SK-N-SH cells (right). ***p < 0.001.

Previous studies have shown that OTX015 had predominant effects on *MYCN*-ampliﬁed NB cells, and depletion of BRD4 resulted in *MYCN* repression (20). We next examined the effect of ARV-825 on the expression of *MYCN* and *c-Myc*. The basal *MYCN* or *c-Myc* expression status was shown in **Figure 7A** and **Supplementary Figure S5**. As expected, the transcript level of *MYCN* and *c-Myc* was drastically decreased in SK-N-BE(2) and SK-N-SH cells in response to BET depletion by ARV-825 (**Figure 7B**). Moreover, the suppression of *MYCN* protein in two *MYCN*-amplified NB cells was both dose- and time-dependent (**Figures 6A**, **7C**). Similarly, in *MYCN* non-amplified NB cells which distinctly express *c-Myc*, ARV-825 downregulates *c-Myc* expression as well (**Figures 6B**, **7D**). Thereby, our observations reveal that ARV-825 perturbs BRD4-mediated *MYCN* and *c-Myc* transcription, leading to *MYCN* and *c-Myc* protein reduction.

### ARV-825 Represses the Expression of *MYCN*-Associated Super Enhancer Genes

Stegmaier K. group has previously identified a set of super-enhancer associated transcription factors which forms a transcriptional core regulatory circuitry (CRC) that determines cell state in *MYCN*-amplified NBs (34). We thus performed real-time PCR to determine whether ARV-825 can influence the transcript levels of these *MYCN*-associated super enhancers, including *ISL1, PHOX2B, HAND2, GATA3*, and *TBX2* in NB cells. In SK-N-BE(2) cells, compared with parental cells, the expression level of each *MYCN*-associated super enhancer gene was dramatically repressed following treatment with ARV-825 (**Figure 7E** left). Even though SK-N-SH cells do not harbor *MYCN* amplification, similar inhibitory effect on those *MYCN*-associated super enhancers was observed as a result of BRD4 depletion, implying that these genes were also regulated by *c-Myc* (**Figure 7E** right). These data provide evidence that ARV-825 downregulates the *Myc*-associated CRC transcription by interfering with BRD4 function, thereby prompting NB cell proliferation suppression.

### ARV-825 Has a Potent Antitumor Effect in Neuroblastoma Xenograft Mouse Model

**Figure 8:**
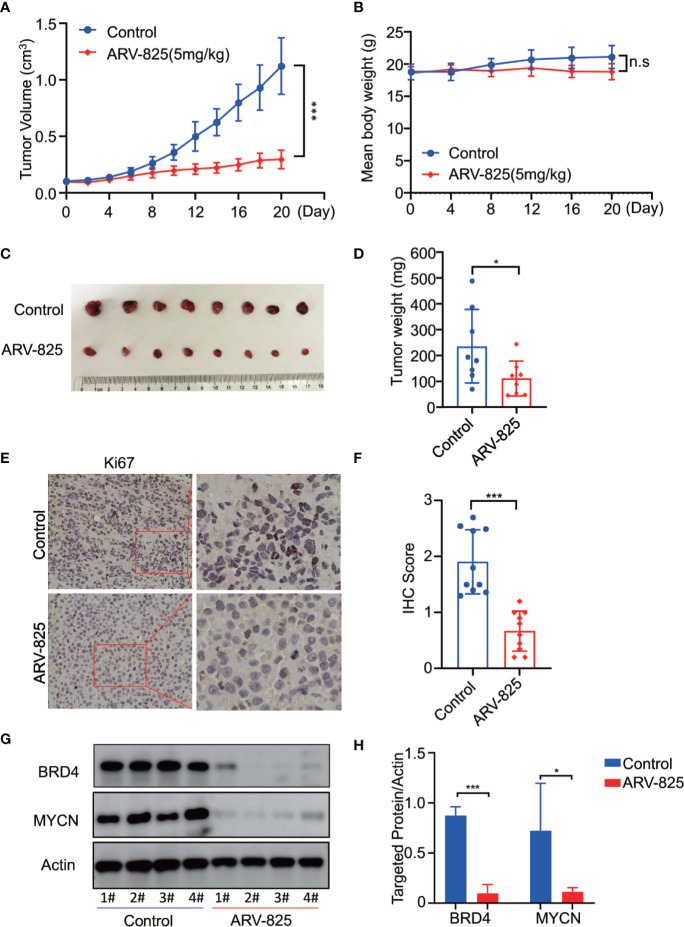
ARV-825 displays anti-tumor efficacy in the NB xenograft model. Nude mice bearing SK-N-BE(2) xenograft tumors were treated by either 5 mg/kg ARV-825 or vehicle control intraperitoneally every day for 20 days. Data are mean ± SEM (n = 8). **(A)** Tumor volume was recorded every 2 days and calculated using the formula: (width × length × height)/2. **(B)** Mice body mass was weighed every 4 days. **(C)** Photograph of xenograft tumors from ARV-825- or vehicle-treated mice. **(D)** Tumor weight from ARV-825- or vehicle-treated mice. **(E)** IHC staining of Ki67 in xenograft tumors from ARV-825- or vehicle-treated mice. **(F)** Scoring results of Ki67 staining in tumors from ARV-825- or vehicle-treated mice. The total scoring (TS) = percentage of positive cells (P) x the intensity (I). **(G)** Western blot analysis of MYCN and BRD4 expression in tumors from ARV-825- or vehicle-treated mice. **(H)** ARV-825 suppressed BRD4 and MYCN protein expression in xenografted tumors from ARV-825- or vehicle-treated mice (Intensity calculated from western blot result). *p < 0.05; ***p < 0.001; n.s, not significant.

To further investigate the *in vivo* activity of ARV-825, we developed the pre-clinical model of neuroblastoma using the *MYCN*-amplified SK-N-BE(2) cell. Five mg/kg ARV-825 was administrated daily when the subcutaneous tumor reached a size of 100 mm^3^. A significant reduction in tumor burden was observed in mice with ARV-825 treatment group compared to those in the control group (**Figure 8A**). The xenograft tumor weight was reduced in mice receiving ARV-825 treatment, but no significant difference in mice body weight was observed between the treatment and control group (**Figures 8B–D**). The proportion of Ki67 positive cells was much lesser in tumors from ARV-825-treated mice (**Figures 8E, F**), indicating a reduction in proliferative activity. Besides that, ARV-825 treatment downregulated the BRD4 and *MYCN* protein expression in ARV-825-treated xenograft tumors than in the control group (**Figures 8G, H**), which is consistent with the *in vitro* results. These observations suggest that ARV-825 can effectively suppress tumor growth in the subcutaneous NB xenograft model.

### Different BRD4 PROTAC Inhibitors Exhibit Anti-NB Activity as ARV-825

Three different PROTAC BRD4 inhibitors (MZ1, dBET1, GNE-98) were used to evaluate their efficacy in neuroblastoma cells. Each PROTAC BRD4 inhibitor was designed based on different E3 ligases and BRD4 inhibitors. MZ1 is a Von Hippel-Lindau tumor suppressor (VHL)-based PROTAC BET inhibitor containing the BRD4 inhibitor JQ1 (35). dBET1 was designed using JQ1 and thalidomide as the ligands of BRD4 and CRBN, respectively (36). GNE-987 was a newly developed chimeric BRD4 degrader with VHL-binding moiety and a potent tetracyclic BRD4 inhibitor (37, 38).

**Figure 9:**
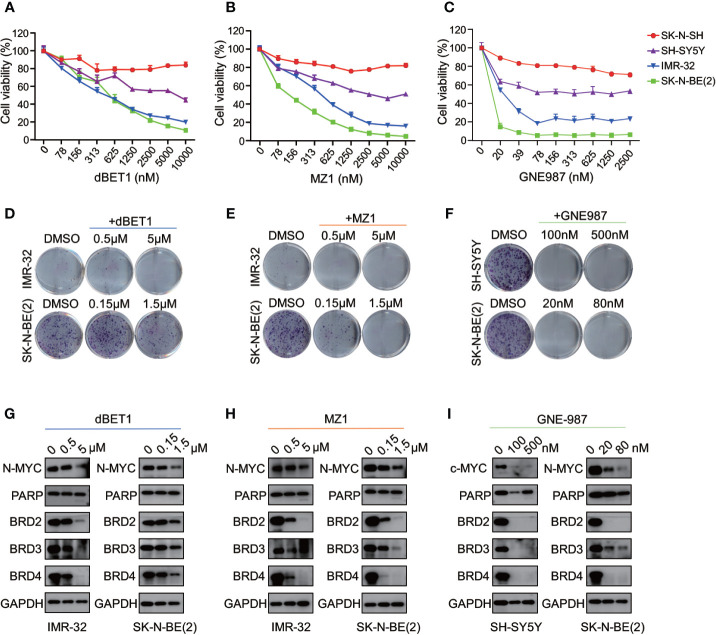
BRD4 PROTAC inhibitors suppressed proliferation in NB cells. CCK8 assay showed that cell proliferation rates were reduced in NB cells treated with serial concentration of dBET1 **(A)**, MZ1 **(B)**, and GNE987 **(C)** for 72 h. Clone formation ability was reduced in NB cells after 7–14 days of dBET1 **(D)**, MZ1 **(E)**, and GNE987 **(F)** treatment. Western blot showed BRD4 PROTAC inhibitors dBET1 **(G)**, MZ1 **(H)**, and GNE-987 **(I)** depleted BRD2, BRD3, and BRD4 protein, repressed MYCN or c-Myc expression and induced PARP cleavage in NB cells.

We treated four NB cells with different concentrations of BRD4 PROTAC inhibitors. Each inhibitor suppressed cell growth in all four NB cells (**Figures 9A–C**). The IC50 of each inhibitor was listed in **Figure S6**. The clonal formation assay showed long-term inhibitory effect of PROTAC BRD4 inhibitors on NB cell proliferation (**Figures 9D–F**). As expected, BRD4-targeted PROTAC inhibitors triggered apoptosis in all four NB cell lines (**Figure S7**). Reduction of BRD2, BRD3, and BRD4 protein was observed in a dose-dependent manner in NB cells treated with each PROTAC BD4 inhibitor (**Figures 9G–I**). In addition, BET inhibitors treatment-induced MYCN and c-Myc protein suppression was seen in all the cells (**Figures 9G–I**). These observations were consistent with the results in NB cells treated with ARV-825.

Altogether, these data from 4 different BRD4 PROTAC inhibitors indicate that PROTAC BRD4 inhibitors suppress *MYCN* or *c-Myc* expression by BET proteins depletion, thus inhibiting cell proliferation and inducing apoptosis in neuroblastoma cells.

## Discussion

Dysregulated expression of *Myc* family gene was a hallmark of neuroblastoma. *MYCN* amplification is regarded as an initiating event that drives the development of high-risk neuroblastomas (3). For *MYCN* non-amplified neuroblastoma, *c-Myc* is predominantly responsible for NB tumor progression (39, 40). However, owing to their “undruggable” protein structure, directly targeting *MYC* family members remains unachievable currently (41). Researchers are now focusing on targeting critical mediators of *MYC* transcription. BRD4 plays a pivotal role in the *Myc* transcription function. Localized at the super enhancer elements loci proximal to *MYC*, BRD4 efficiently facilitates *Myc* expression, as well as *Myc*-driven transcription. Thereby, targeting BRD4 could be an alternative approach for targeting *Myc*-driven tumors. Although our results showed that BRD4 expresses universally in neuroblastoma cells as well as a variety of other types of tumors, high expression of BRD4 was closely related to poor prognostic outcome in neuroblastoma, suggesting BRD4 can potentially serve as a prognostic marker in neuroblastoma.

In the recent years, there is an emergence of using PROTAC technology to target conventionally undruggable cancer targets. Compared with traditional small molecule inhibitors, PROTAC inhibitors can achieve better inhibitory effect by specific and sustained depletion of a protein of interest through proteasome-dependent degradation. ARV-825, a novel BET inhibitor using PROTAC technology, has shown promising preclinical efficacy in multiple types of tumors, such as AML, MM, HCC, and liposarcoma (30, 42, 43). Given the advantage of ARV-825 over the traditional BETi, we evaluated the anti-cancer effect of ARV-825 in pediatric neuroblastoma. Our results showed the potent anti-proliferative activity of NB cells to ARV-825, with IC50 in the nanomolar range. Furthermore, ARV-825 inhibited cell cycle progression and prompted pro-apoptotic response. These results are consistent with previous studies of the anti-tumor activity of ARV-825 *in vitro*.

Previous studies have shown that the potency of BRD4 inhibitors, such as JQ1 and OTX-015, is dependent on the *MYCN* status (8, 20). However, for OTX-015, there is a poor correlation between IC50 values and both *MYCN* mRNA and protein levels in NB cells (20). In the current study, strong correlation between *MYCN* status and sensitivity to ARV-825 was not observed. It is likely that this phenomenon results from the limited number of cell lines we used in this study. Only two *MYCN*-amplified NB cells was used to determine the anti-tumor activity of ARV-825. IMR-32 showed higher sensitivity to ARV-825 than the other two *MYCN* non-amplified NB cells. However, the dependency on *MYCN* status was not observed in SK-N-BE(2) cells. The difference between these two cell lines may due to the *MYCN* copy number variation, leading to different impact of ARV-825 on *MYCN* expression. In IMR-32, MYCN protein was reduced at 1nM of ARV-825 treatment. By contrast, in SK-N-BE(2) cells, which harbored a higher *MYCN* copy number and *MYCN* expression, significant *MYCN* protein suppression was observed at 1 μM. In mRNA level, only 50% of *MYCN* transcript was suppressed even at 500 nM and 1 μM of ARV-825 treatment in SK-N-BE(2) cells. This partial suppression of *MYCN* may confer survival advantage and drug resistant in SK-N-BE(2) cells, leading to a relatively high IC50 than other cells.

CRBN is an E3 ligase that is widely employed in PROTAC technology. By recruiting the targeted molecules, CRBN efficiently facilitates the degradation of protein of interest. Previous studies have demonstrated that CRBN expression level as a predictive marker to ARV-825 efficacy (43). Our finding indicates that CRBN expression plays an essential role in the sensitivity to ARV-825 in NB cells, as shRNA-mediated knockdown of CRBN expression reduced the sensitivity to ARV-825 in NB cells. Mechanistically, previous study showed that PROTAC inhibitors exerts their effects by forming POI (protein of interest)-PROTAC-E3 ternary complex, and subsequently degrades the POI by proteasome (44). Silencing the expression of CRBN will disrupt BRD4-ARV-825-CRBN complex formation, and decrease BRD4 degradation activity, which will result in a reduced sensitivity to ARV-825. However, despite a relatively low CRBN expression, the IMR-32 cell line is most sensitive to ARV-825 among all 4 NB cell lines. One possible explanation was IMR-32 cells harbors *MYCN* amplification, which was reported to be more sensitive to BRD4 inhibitor than *MYCN* non-amplified cell lines. It is also reasonable to speculate that the loss of CRBN in cells might result in CRBN-based PROTACs resistance. In that case, other PROTAC molecules which employed an alternative E3 ligase should be used (24).

The role of ARV-825 as a BRD4 degrader is confirmed by our findings that BRD4 protein expression was reduced after ARV-825 treatment. Moreover, a decrease in both BRD2 and BRD3 proteins were also observed. Similar findings were reported in other studies using BETi (29, 45, 46). This phenomenon could be explained that OTX015, which is part of the ARV-825 structure, can bind to all BET family owing to the high homologue domains in BET family members (47). Interestingly, it is also reported that in some cases BRD2 protein accumulated after exposure to OTX015, which could be a compensatory mechanism in response to depletion of other BET protein members (48, 49). ARV-825 caused profound depletion of BRD2 and BRD4 as distinct from OTX015 (49). Further investigation is required to determine the different influences and intrinsic mechanism of BET inhibitors on the BET family proteins expression.

BRD4 is an established Myc regulator. Several independent reports have shown that BETi displaces the BRD4-chromatin interaction, thereby repressing *MYC* and *MYC* target gene expression (8, 20, 50). Our results also implicate that the pharmacologic inhibition of BRD4 by ARV-825 caused a reduction in *MYCN* or *c-Myc* mRNA and protein expression in neuroblastoma. In *MYCN*-amplified NB, *MYCN* was reported to form CRC with a set of essential transcription factors which act as a network in a feed-forward, autoregulatory manner (34). We further showed that in addition to *MYCN*, each member of the *MYCN*-amplified NB-specific CRC was strikingly inhibited by ARV-825. Similarly, a reduced expression was found in *MYCN* non-amplified NB cells, suggesting this CRC also exists in *MYCN* non-amplified NB.

In the neuroblastoma xenograft model, we showed that ARV-825 inhibited the SK-N-BE(2) xenograft tumor growth. In consistence with *in vitro* results, ARV-825 treatment downregulated BRD4 and MYCN protein expression in xenograft tumor. This further validates the effectiveness of ARV-825 in blocking the BRD4-MYCN pathway. It is important to note that although mice treated with ARV-825 has less body weight gain as compared to the control group, the difference is not statistically significant. Recent evidence has suggested that mice treated with JQ1 or mice with partial loss of BRD4 have impaired adipogenesis capability, thus resulting in a decrease in body weight (51). Apart from the bodyweight, no obvious toxic effect was found in organs from ARV-825 treated mice (data not shown).

In conclusion, our results demonstrate that the PROTAC BET inhibitor ARV-825 has potent anti-tumor activity in neuroblastoma both *in vitro* and *in vivo*. ARV-825 exerts its effect by efficiently degrading BET proteins, leading to *MYCN* and *c-Myc* suppression. Our studies show that ARV-825 is a novel therapeutic approach for neuroblastoma treatment.

## Data Availability Statement

All datasets presented in this study are included in the article/**Supplementary Material**.

## Ethics Statement

The animal study was reviewed and approved by Animal Care and Use Committee at Children’s hospital of Soochow University.

## Author Contributions

JP and SH designed and directed the study. SX helped statistical analysis and manuscript draft. ZL, YT, and XL performed most of the experiments. SL performed lentivirus preparation and transfection. ZZ and XZ participated in Western blotting, PCR and the *in vitro* experiments. CY and ML participated in establishing the neuroblastoma xenograft model. XC and YW did the IHC staining. YX and FF supported the design of primers for real-time PCR. YL helped with the apoptosis and cell cycle analysis. GQ participated in plasmid construction. All authors contributed to the article and approved the submitted version.

## Funding

This work was supported by grants from the National Natural Science Foundation (82072767, 81770145, 81702339, 81701596, 81802499, 81872845, 81902534); Natural Science Foundation of Jiangsu Province (BK20180207, BK20180206, SBK2019021442, BK20191175, BK20190186); Jiangsu province’s science and technology support program (Social Development) project (BE2017658; BE2017659); The 333 High-level Personnel Training Project of Jiangsu Province (BRA2016530); Jiangsu Provincial Medical Talent (Jian Pan); “Six Talent Peak” High-level Talent Project (2016-WSN-129); the Universities Natural Science Foundation of Jiangsu Province (No.16KJB310014); Jiangsu Government scholarship for overseas studies program (JS-2018-124); Jiangsu Provincial Medical Youth Talent (No.QNRC2016762, QNRC2016756,QNRC2016768); Gusu Health Talents program of Soochow city (2020-104); Department of Pediatrics Clinical Center of Suzhou (Szzx201504); the Applied Foundational Research of Medical and Health Care of Suzhou City (SYS2018075, SYS2018074, SYS2018074, SYS2019078, SS201709, SS201809).

## Conflict of Interest

The authors declare that the research was conducted in the absence of any commercial or financial relationships that could be construed as a potential conflict of interest.

## Abbreviations

NB, Neuroblastoma; BET, Bromodomain and extra terminal; BRD4, Bromodomain-containing protein 4; CCK-8, Cell counting kit-8; CRC, Core regulatory circuitry; IC50, 50% of maximal inhibitory concentration; IHC, Immunohistochemistry; PBS, Phosphate-buffered saline; PCR, Polymerase chain reaction; shRNA, Short hairpin RNA.
