# Supplementary material for: PROTAC Bromodomain Inhibitor ARV-825 Displays Anti-Tumor Activity in Neuroblastoma by Repressing Expression of MYCN or c-Myc
Source: Front Oncol. 2020 Nov 26;10:574525. doi: 10.3389/fonc.2020.574525 (PMC7726414; doi:10.3389/fonc.2020.574525)
Supplement: Supplementary file 1 [file Presentation_1.pdf]

Supplement material

1. Sequence

**CRBN shRNA sequence**		
CCGGGCCCACGAATAGTTGTCATTTCTCGAGAAATGACAACTATTCGTGGG		
**Primers used in real-time PCR**		
Gene name	Direction	Primer sequence (5’→3’)
BRD2	F	CCGTCGAACTCAAGTACAATGG
	R	GCTCTGGCGATTGAAAGGTGT
BRD3	F	TCAAATTGAACCTGCCGGATT
	R	TGCATACATTCGCTTGCACTC
BRD4	F	ACCTCCAACCCTAACAAGCC
	R	TTTCCATAGTGTCTTGAGCACC
CRBN	F	TCCTTGAGCTAAGAACACAGTCA
	R	AAGGCAACACACATTCGGGAA
MYCN	F	CACAGTGACCACGTCGATTT
	R	CACAAGGCCCTCAGTACCTC
c-Myc	F	TCCCTCCACTCGGAAGGAC
	R	CTGGTGCATTTTCGGTTGTTG
HAND2	F	CAGCAACGACAAGAAAACCA
	R	GGATGATTCCAAATGCAAGG
ISL1	F	GGCATGTTTGAAATGTGCGG
	R	ACACAGCGGAAACACTCGAT
PHOX2B	F	CTTCGCGGAGACTCACTACC
	R	CTCCTGCTTGCGAAACTTGG
GATA3	F	TTCAGTTGGCCTAAGGTGGT
	R	CGCCGGACTCTTAGAAGCTA
TBX2	F	GGCCTTCCACAAGCTGAAG
	R	GCGGCTGGTACTTGTGCAT
β-actin	F	AGCGAGCATCCCCCAAAGTT
	R	GGGCACGAAGGCTCATCATT

2. Supplement Figure S1

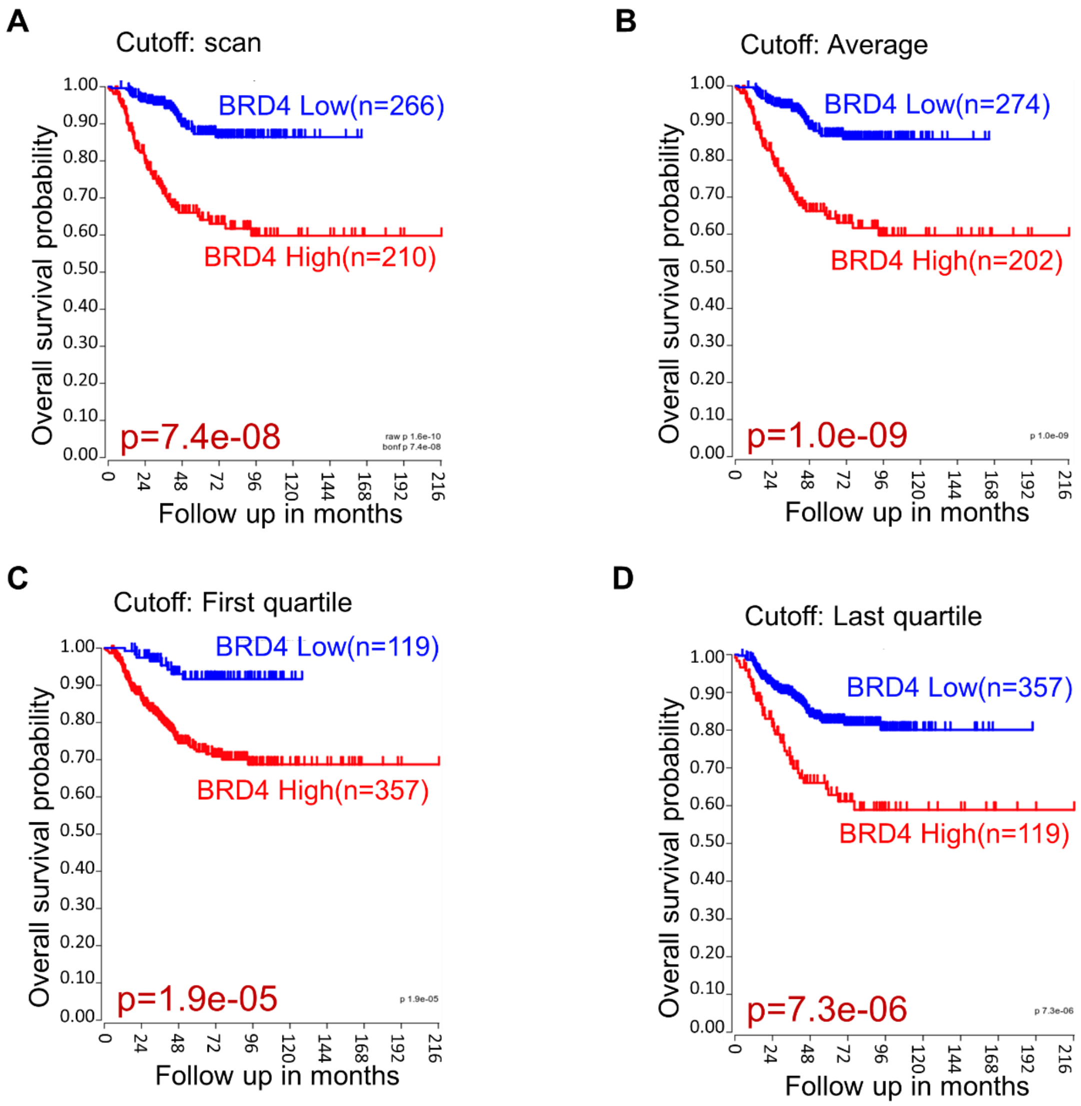

Supplement Figure S1 High BRD4 indicates poor prognosis of NB patients.

Kaplan-Meier plots generated in R2 platform using Kocak cohort (including 649 NB samples) by applying cutoff value as Scan (A), Average (B), First Quartile (C), and Last Quartile (D) modi.

3. Supplement Figure S2

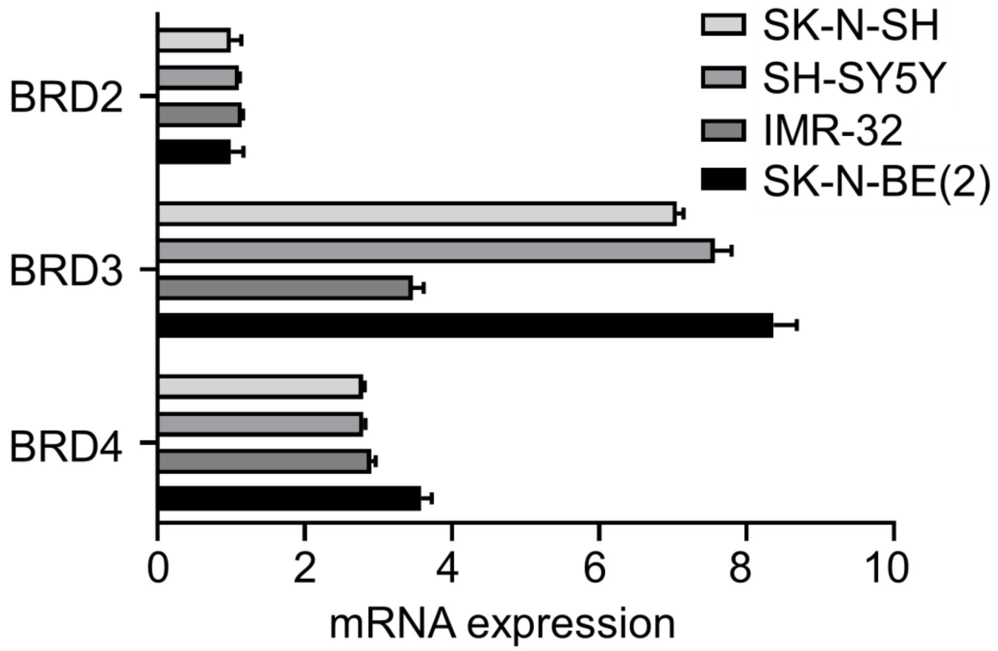

Supplement Figure S2 mRNA expression level of BET family members in NB cells.

4. Supplement Figure S3

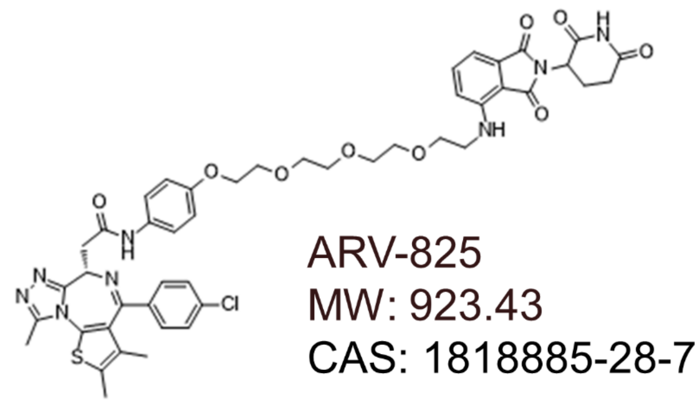

Supplement Figure S3 Chemical structure of ARV-825.

5. Supplement Figure S4

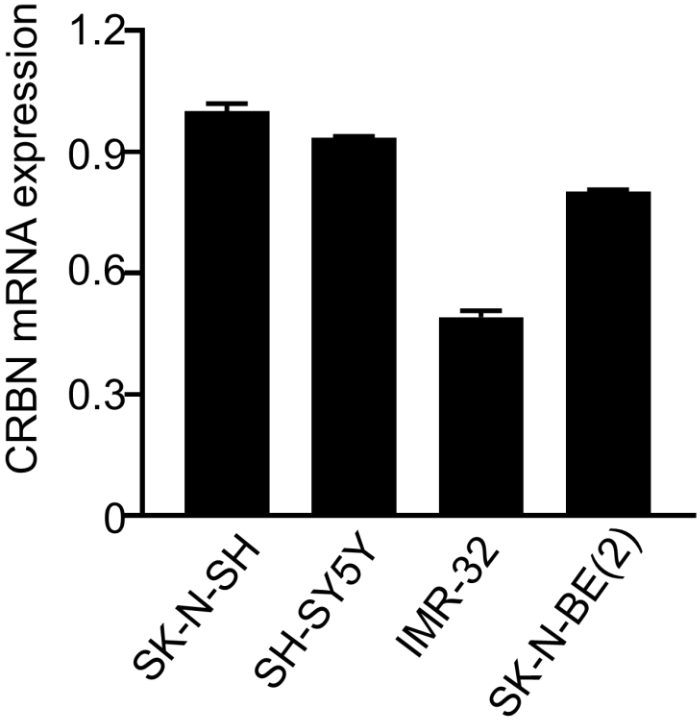

Supplement Figure S4 *CRBN* mRNA expression level in NB cells.

6. Supplement Figure S5

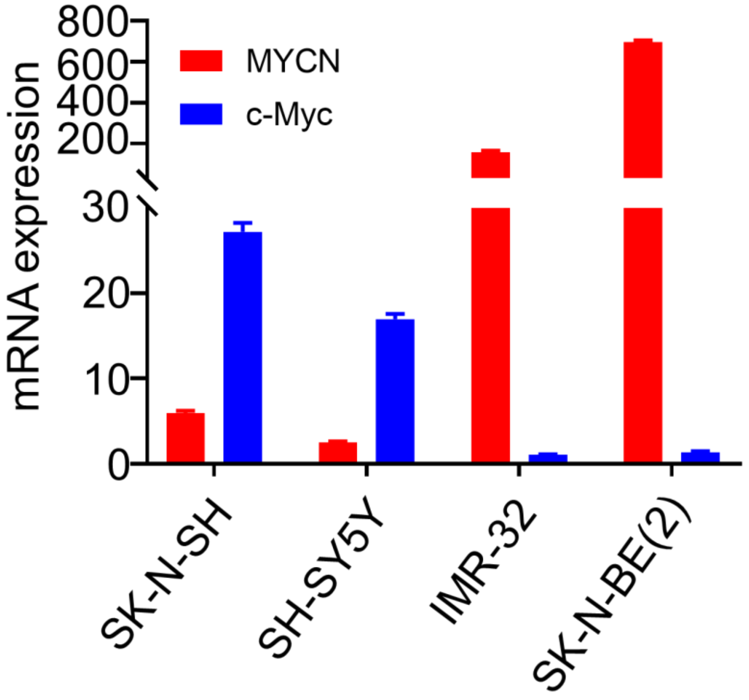

Supplement Figure S5 *MYCN* and *c-Myc* mRNA expression level in NB cells.

7. Supplement Figure S6

Cell line	dBET1	MZ1	GNE987
SK-N-SH	>10μM	>10μM	>10μM
SH-SY5Y	>10μM	>10μM	2.49μM
IMR-32	518.4nM	431.4nM	0.27nM
SK-N-BE(2)	561.0nM	119.8nM	4.440e-006nM

Supplement Figure S6 IC50 of PROTAC BRD4 inhibitors in NB cells.

8. Supplement Figure S7

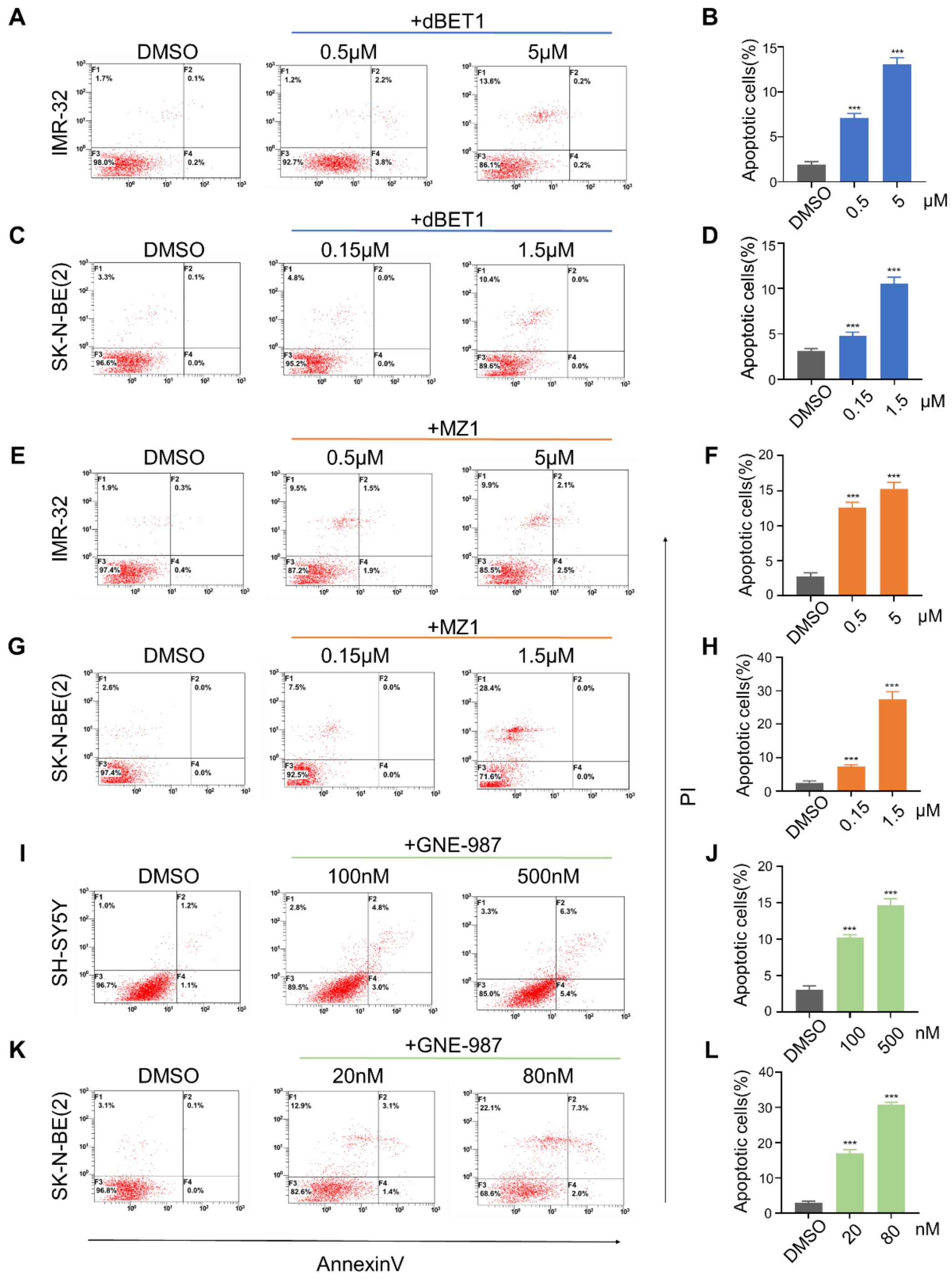

Supplement Figure S7 PROTAC BRD4 inhibitors induces apoptosis in NB cells.

Annexin V/PI staining showed 72h of PROTAC BRD4 inhibitors treatment induced apoptosis in NB cells.
